# Multicenter phase II study of docetaxel plus oxaliplatin combination chemotherapy in patients with advanced gastric cancer: Daegu Gyeongbuk Oncology Group

**DOI:** 10.1038/sj.bjc.6604188

**Published:** 2008-01-22

**Authors:** J G Kim, S K Sohn, Y S Chae, H S Song, K-Y Kwon, Y R Do, M K Kim, K H Lee, M S Hyun, H M Ryoo, S H Bae, K U Park, W S Lee, J H Baek, H Y Chung, W Yu

**Affiliations:** 1 Department of Oncology/Hematology, Kyungpook National University Hospital, Kyungpook National University School of Medicine, Daegu, Korea; 2 Department of Hematooncology, Dongsan Medical Center, Keimyung University School of Medicine, Daegu, Korea; 3 Department of Oncology/Hematology, Yeungnam University Hospital, Daegu, Korea; 4 Department of Oncology/Hematology, Daegu Catholic University Medical Center, Daegu Catholic University School of Medicine, Daegu, Korea; 5 Department of Oncology/Hematology, Dongguk University Medical Center, Dongguk University College of Medicine, Gyeongju, Korea; 6 Department of Oncology/Hematology, Daegu Fatima Hospital, Daegu, Korea; 7 Department of Oncology/Hematology, Ulsan University Hospital, Ulsan, Korea; 8 Department of General Surgery, Kyungpook National University Hospital, Kyungpook National University School of Medicine, Daegu, Korea

**Keywords:** docetaxel, oxaliplatin, chemotherapy, gastric cancer

## Abstract

The present study was conducted to evaluate the efficacy and safety of a combination regimen of docetaxel plus oxaliplatin in patients with advanced gastric cancer. Patients with previously untreated metastatic or recurrent, measurable gastric cancer received intravenous docetaxel 65 mg m^−2^ plus oxaliplatin 120 mg m^−2^ on day 1 based on a 3-week cycle. Forty-two patients were enrolled in the current study, among whom 39 were assessable for efficacy and all assessable for toxicity. One complete response and 18 partial responses were confirmed, giving an overall response rate of 45.2% (95% confidence interval (CI); 31.7–59.7%). At a median follow-up of 7.7 months, the median time to progression and median overall survival was 5.7 (95% CI; 4.3–7.2) months and 9.9 (95% CI; 7.8–12.0) months, respectively. Grade 3/4 neutropenia occurred in 11 patients (26.1%) and febrile neutropenia was observed in four patients (9.5%). The common non-haematologic toxicity was fatigue (grade 1/2, 61.9%) and nausea (grade 1/2, 47.7%). The combination of docetaxel and oxaliplatin was found to be well tolerated and effective in patients with advanced gastric cancer.

Although the prognosis for advanced gastric cancer is poor, combination chemotherapy has improved the quality of life and overall survival compared with the best supportive care in several randomised studies (Murad *et al*, 1993; Glimelius *et al*, 1994; Pyrhonen *et al*, 1995). Among the various active chemotherapeutic agents, cisplatin-based combination chemotherapy has been most commonly used with a high response rate of 37–56% (Kim *et al*, 1993; Boku *et al*, 1999; Roth *et al*, 2000; Vanhoefer *et al*, 2001).

Docetaxel, a semi-synthetic taxoid developed in the eighties, is derived from the needles of the European yew tree, *Taxus baccata* (Pazdur *et al*, 1993), and phase II studies have shown that docetaxel, alone or in combination with cisplatin, is active against advanced gastric cancer (Sulkes *et al*, 1994; Roth *et al*, 2000; Bang *et al*, 2002). Furthermore, the additive effect of docetaxel in combination with 5-flourouracil/cisplatin for the treatment of advanced gastric cancer was also reported in a randomised phase III study (Van Cutsem *et al*, 2006).

Oxaliplatin is a third-generation platinum derivative, the activity of which is based on the formation of DNA adducts that inhibit replication and transcription. However, the adducts of oxaliplatin appear to be more effective than cisplatin adducts with regard to the inhibition of DNA synthesis (Schmidt and Chaney, 1993; Mamenta *et al*, 1994; Woynarowski *et al*, 2000). In clinical studies, oxaliplatin has also shown antitumour activity when given as a monotherapy or in combination with 5-fluorouracil in patients with various solid tumours including gastric cancer (Louvet *et al*, 2002; Al-Batran *et al*, 2004; Cassidy *et al*, 2004; Suh *et al*, 2005; Park *et al*, 2006). In comparison with cisplatin, oxaliplatin exhibits a favourable toxicity profile with a substantially lower rate of nephrotoxicity, ototoxicity, and myelosuppression (Extra *et al*, 1990; Misset *et al*, 2001; Louvet *et al*, 2002; Al-Batran *et al*, 2004; Suh *et al*, 2005; Park *et al*, 2006).

Since these two active agents have different anticancer mechanism and little overlap in terms of their key side effects except neurotoxicity, docetaxel, and oxaliplatin would appear to be a good combination regimen. As such, in recent clinical studies, the combination of docetaxel and oxaliplatin demonstrated active antitumour effect and manageable toxicity profile in patients with lung cancer or ovarian cancer (Kouroussis *et al*, 2003; Raez *et al*, 2006; Ferrandina *et al*, 2007). However, there is no published data on the efficacy of a combination of docetaxel and oxaliplatin as a first-line treatment for advanced gastric cancer.

Therefore, we conducted a phase II study to evaluate the response rate, time to progression, duration of response, overall survival, and safety of a combination regimen of docetaxel plus oxaliplatin in patients with advanced gastric cancer. The schedule and doses of docetaxel and oxaliplatin in the present study were determined according to previous dose finding and phase II study (Kouroussis *et al*, 2003; Raez *et al*, 2006).

## PATIENTS AND METHODS

### Eligibility

All the patients involved in the current study had histologically confirmed metastatic or recurrent gastric adenocarcinoma with at least one unidimensionally measurable lesion. (i.e., a diameter ⩾1 cm, as assessed by spiral computed tomography). The patients were 19–70 years of age with a performance status of 0–2 on the Eastern Cooperative Oncology Group (ECOG) scale. Plus, adequate haematological (absolute neutrophil count ⩾1.5 × 10^9^ l^−1^, platelet count ⩾100 × 10^9^ l^−1^, haemoglobin ⩾9 g dl^−1^), renal (serum creatinine ⩽1.5 mg dl^−1^ and creatinine clearance ⩾50 ml min^−1^), and hepatic (total bilirubin⩽1.5 mg dl^−1^ and serum transaminase level⩽2 times the upper limit of the normal range) levels were also required. Patients who had received adjuvant chemotherapy completed 4 weeks before entry were eligible. Patients were ineligible if they had previously received palliative chemotherapy or radiation therapy, or had other severe medical illnesses, central nervous system metastasis, severe peripheral neuropathy, another active malignancy, or history of anaphylaxis to drugs. The institutional review board of each author's institution approved the protocol, and written informed consent was obtained from all patients before enrolment.

### Study treatment

The docetaxel 65 mg m^−2^ was administered through a 1-h intravenous infusion on day 1. The oxaliplatin (Oxalpla®, Yuhan Co, Seoul, Korea) 120 mg m^−2^ was also administered after the docetaxel infusion over 2-h on day 1. All patients were premedicated with a dexamethasone (8 mg b.i.d. from 1 day before to 1 day after docetaxel infusion) to prevent hypersensitivity reactions of docetaxel. Antiemetic treatment was routinely given before each cycle of chemotherapy. The prophylactic use of a colony-stimulating factor (CSF) was not permitted in the first cycle. Chemotherapy was given every 3 weeks and continued until disease progression, patient refusal, or an unacceptable toxicity up to 9 cycles.

### Dose modification

The next course of treatment began only when the neutrophil count was ⩾1.5 × 10^9^ l^−1^, the platelet count was ⩾75 × 10^9^ l^−1^, and any other treatment-related toxicities were less than or equal to grade 1; otherwise, treatment was withheld for up to 2 weeks. If adverse events did not improve to grade 0 or 1 after 3 weeks, the patients were excluded from the study. Treatment was continued at the same dose if patients experienced grade 1 toxicities or other toxicities considered by the investigator unlikely to become serious or life threatening (e.g., alopecia). For all other treatment-related adverse events with a grade 2 intensity or higher, the dose modification scheme described below was implemented. No dose reduction was required after the first appearance of a grade 2 toxicity, although treatment was interrupted until the toxicity was resolved to grade 0–1. The doses of docetaxel and oxaliplatin were reduced by 20% in patients who experienced a second occurrence of a given grade 2 toxicity or any grade 3 toxicity. If patients experienced a third occurrence of a given grade 2 toxicity, a second occurrence of a given grade 3 toxicity, or any grade 4 toxicity, both doses were reduced by 40%. The docetaxel and oxaliplatin were both discontinued if, despite a dose reduction, a given toxicity occurred for a fourth time at grade 2, a third time at grade 3, or a second time at grade 4. If adverse events did not improve to grade 0 or 1 after 3 weeks, the patients were excluded from the study.

### Study assessments

A screening assessment, including a medical history, physical examination, ECG, chest X-ray, and tumour assessment was conducted within 2 weeks before starting treatment. Further assessments conducted within 7 days before starting treatment included vital signs, an ECOG performance status, and laboratory tests. Complete blood counts were performed weekly during the first cycle and every cycle thereafter, and biochemical tests performed before each cycle. Tumours were measured every two cycles until the tumour progressed. The tumour responses were classified according to the response evaluation criteria in solid tumours guidelines (Therasse *et al*, 2000). Patients with a complete response or partial response required a confirmatory disease assessment at least 4 weeks later. Adverse events were graded according to National Cancer Institute Common Toxicity Criteria version 3.0. Peripheral sensitive neuropathy was graded according to the oxaliplatin-specific scale reported by previous study (Caussanel *et al*, 1990). Dose intensity was defined as the total amount of drug given (mg m^−2^) divided by the number of weeks.

### Statistical analysis

The current trial used a two-stage optimal design, as proposed by Simon, with a 90% power to accept the hypothesis and 5% significance to reject the hypothesis (Simon, 1989). Plus, the current trial was designed to detect a response rate of 45% as compared to a minimal, clinically meaningful response rate of 20%. Allowing for a follow-up loss rate of 10%, the total sample size was 42 patients with a measurable disease. All enrolled patients were included in the intention-to-treat analysis of efficacy. The duration of response, time to progression (TTP), and survival analyses were all estimated using the Kaplan–Meier method. The duration of response was defined as the interval from the onset of a complete response or partial response until evidence of disease progression was found. Meanwhile, the TTP was calculated from the initiation of chemotherapy to the date of disease progression, while overall survival was measured from the initiation of chemotherapy to the date of the last follow-up or death. The statistical data were obtained using an SPSS software package (SPSS 11.0 Inc., Chicago, IL, USA).

## RESULTS

### Patient characteristics

From February to November 2006, a total of 42 patients were enrolled in the current study from six centers. The characteristics of the patients are summarised in Table 1. The median age was 58 (range, 29–70) years, with 33 males and nine females. Most of the patients (85.7 %) had a good performance status (ECOG 0 or 1). Thirty (71.4 %) patients had a metastatic disease, while 12 patients had a recurrent disease after surgical resection (total or subtotal gastrectomy) of the primary tumour. Distant lymph nodes and the liver were the most common sites of the metastases. No patients had received prior palliative chemotherapy or radiotherapy.

### Efficacy

**Figure 1:**
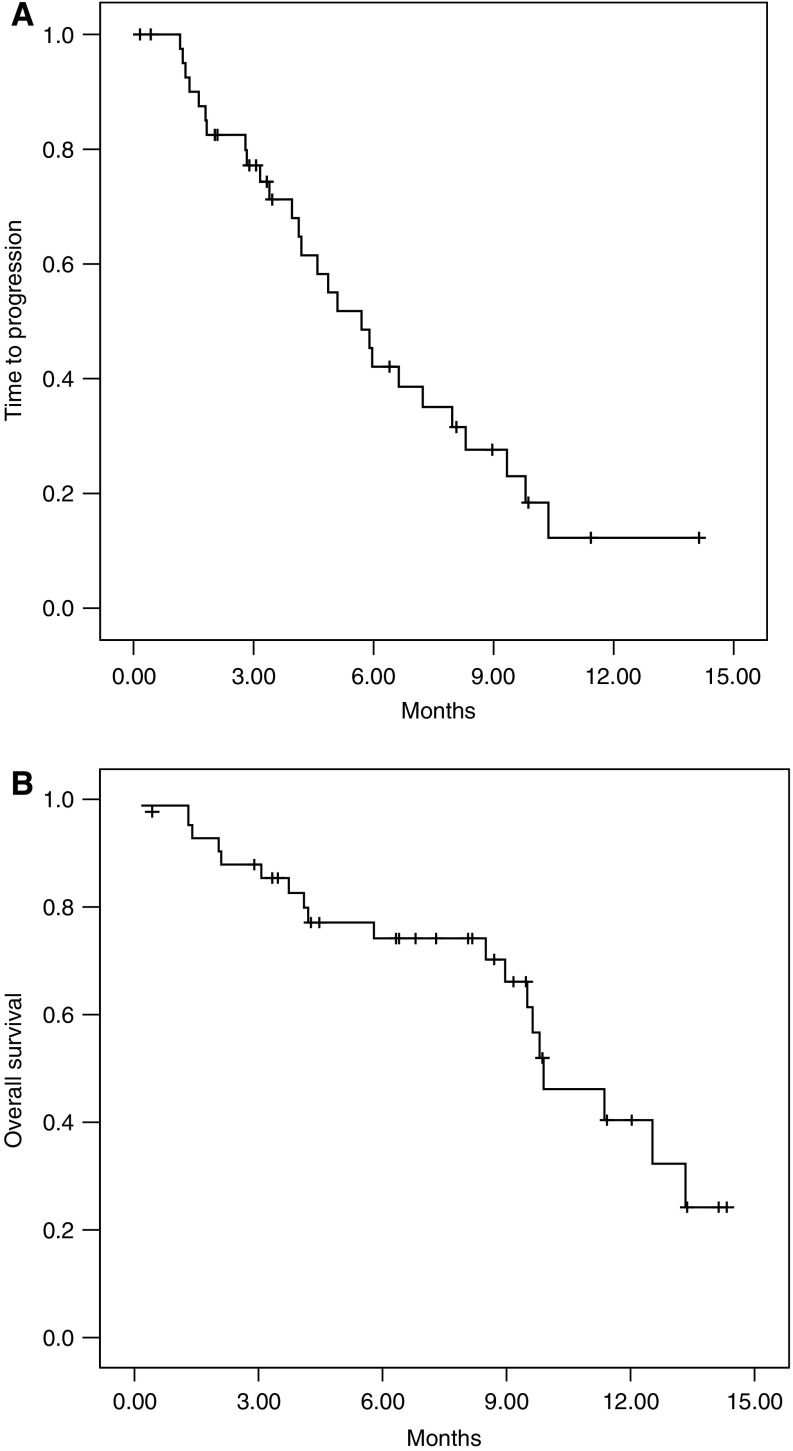
Kaplan–Meier curves for time to disease progression (**A**) and overall survival (**B**) for intention-to-treat population (*n*=42). The median time to progression and median overall survival was 5.7 and 9.9 months, respectively.

Thirty-nine (92.9%) of the 42 patients were assessable for response, with the remaining three being lost to follow-up or patient refusal. All efficacy data are reported using the intent-to-treat patient population. One case of complete response and 18 cases of partial response were confirmed, giving an overall response rate of 45.2% (95% confidential interval [CI]; 31.7–59.7%). The response characteristics are shown in Table 2. The median duration of response in the 19 responding patients was 6.2 (95% CI; 4.8–7.6) months, while the median TTP for all patients was 5.7 (95% CI; 4.3–7.2) months at a median follow-up duration of 7.7 (range, 0.2–14.1) months (Figure 1). Twenty-five patients (59.5%) received a second-line therapy, such as irinotecan, capecitabine, S-1, or cisplatin after disease progression. Nineteen patients had died at the time of the present evaluation. The estimated median overall survival was 9.9 (95% CI; 7.8–12.0) months with an estimated 1-year survival rate of 40.4±10.5% (Figure 1).

### Toxicity

The haematologic and non-haematologic toxicities that occurred during the current study are summarised in Table 3. A total of 186 cycles (median 4; range: 1–9 cycles) were administrated in 42 patients assessable for toxicity. The most severe haematologic adverse event was neutropenia, which occurred with a grade 3/4 intensity in 11 patients (26.1%) and in 38 cycles (20.4%). Plus, febrile neutropenia was observed in four patients (9.5%). However, all cases were successfully treated with antibiotics and G-CSF. Nausea and fatigue were the most common non-haematologic toxicities. Grade 1/2 nausea and fatigue was observed in 47.7 and 61.9% of patients, respectively. Seventeen (40.4%) patients also experienced a grade 1/2 peripheral neuropathy. Yet, no grade 4 non-haematologic toxicity was observed. Six patients (14.3%) were hospitalised due to treatment toxicities (four due to febrile neutropenia, one due to pneumonia, and one due to general weakness), and one patient died of pneumonia during this study. The dose was reduced in 12 cycles and treatment delayed in nine cycles. The treatment doses were modified for the following reasons: haematologic toxicity (66.6%), neuropathy (16.6%), fatigue (8.3%), and vomiting (8.3%). The mean dose intensity for the docetaxel and oxaliplatin over all treatment cycles was 19.6 mg m^−2^ per week and 37.5 mg m^−2^ per week, corresponding to 90.4 and 93.7% of the planned dose intensities, respectively.

## DISCUSSION

In the current study, the combination chemotherapy of docetaxel and oxaliplatin, which can be administered on an outpatient basis, produced active antitumour activity, and a safe toxicity profile in patients with advanced gastric cancer. The overall response rate (45.2%), median TTP (5.7 months), and median overall survival (9.9 months) following treatment with the present regimen were comparable with previous results reported for cisplatin-based combinations (Boku *et al*, 1999; Roth *et al*, 2000; Vanhoefer *et al*, 2001), where a continuous infusion of a 5-flourouracil (5-FU) and cisplatin regimen achieved a response rate of 51% and median TTP of 5.45 months (Vanhoefer *et al*, 2001), while docetaxel plus cisplatin regimens achieved a response rate and median TTP of 48% and 6.6 months, respectively (Roth *et al*, 2000). However, in a recent meta-analysis of randomised phase II and phase III studies best survival results were achieved with three-drug regimens containing 5-FU, an anthracycline, and cisplatin in patients with advanced gastric cancer (Wagner *et al*, 2006).

Oxaliplatin, which is commonly used in the treatment of colorectal cancer, has also been shown to be active and safe in the treatment of locally advanced or metastatic gastric cancer (Louvet *et al*, 2002; Al-Batran *et al*, 2004; Suh *et al*, 2005; Park *et al*, 2006; Lee *et al*, 2007). A phase II study by Louvet *et al* (2002) reported that an oxaliplatin, fluorouracil, and folinic acid (FOLFOX6) regimen produced a high response rate of 44.9%, and a recent phase II trial (Park *et al*, 2006) on oxaliplatin and capecitabine combination chemotherapy also showed high response rate of 65% and median progression-free survival of 7.5 months in patients with advanced gastric cancer. Furthermore, oxaliplatin was found to be not inferior to cisplatin in a randomised phase III study comparing capecitabine with fluourouracil and oxaliplatin with cisplatin (Cunningham *et al*, 2006). However, oxaliplatin-containing regimens might cause significant peripheral neuropathy. Since there is no effective prophylaxis or treatment for peripheral neuropathy, this can interrupt treatment or reduce the dose intensity of oxaliplatin. For example, severe (grade 3) peripheral neuropathy was observed in 21% of patients and 9.4% of patients discontinued treatment due to neuropathy in the FOLFOX6 regimen (oxaliplatin 100 mg m^−2^ every 2 weeks), although it only occurred in patients benefiting from the treatment, with a median cumulative dose of 901 mg m^−2^ (Louvet *et al*, 2002). Accordingly, the current study used a reduced dose of oxaliplatin (120 mg m^−2^ every 3 weeks) and chemotherapy was given up to maximum 9 cycles, resulting in that no patient experienced grade 3 neuropathy or discontinuation of treatment and the dose intensity of oxaliplatin was 93.7%.

One of the major toxicities related to docetaxel is myelosuppression. Chemotherapy-induced severe neutropenia can also result in treatment-related hospitalisation or mortality, thereby compromising the quality of life and increasing medical expenditure. In a previous study by Roth *et al* (2000) (docetaxel 85 mg m^−2^ and cisplatin 75 mg m^−2^ based on 3-week intervals), grade 3/4 neutropenia was observed in 80% of patients and febrile neutropenia in 16.7% of patients with advanced gastric cancer. In a recent randomised trial reporting a higher response rate and longer overall survival for a triplet of docetaxel, infusional 5-fluorouracil, and cisplatin compared to a doublet of infusional 5-fluorouracil and cisplatin, grade 3/4 neutropenia, and febrile neutropenia also occurred in 84 and 16% of the patients receiving a triplet regimen, respectively (Van Cutsem *et al*, 2006). However, in the current study with a reduced dose of docetaxel (65 mg m^−2^ every 3 weeks), 11 patients (26.1%) experienced grade 3/4 neutropenia and four patients (9.5%) were hospitalised due to febrile neutropenia. Furthermore, in a phase II study by Ferrandina *et al* (2007) that employed docetaxel (75 mg m^−2^) and oxaliplatin (100 mg m^−2^) for the second-line treatment of recurrent ovarian cancer, 32.5% of patient experienced grade 3/4 neutropenia.

In conclusion, docetaxel plus oxaliplatin combination chemotherapy was found to be well tolerated and effective in patients with advanced gastric cancer. However, further studies are warranted to clarify the role of this regimen compared to active triplet regimens in the first-line treatment for advanced gastric cancer.

## Figures and Tables

**Table 1:** Patient characteristics

**Characteristic**	**Number of patients, *n*=42 (%)**
*Age (years)*	
Median (range)	58 (29–70)
Male/female	33 (78.6)/9 (21.4)
	
*ECOG performance status*	
0	5 (11.9)
1	31 (73.8)
2	6 (14.3)
	
*Disease status*	
Metastatic	30 (71.4)
Recurrent	12 (28.6)
	
*Location of primary tumour*	
Upper	6 (14.3)
Middle and lower	36 (85.7)
	
*Metastatic sites*	
Lymph node	18 (35.3)
Liver	16 (30.8)
Peritoneum	9 (17.3)
Ovary	2 (3.8)
Others (lung, adrenal gland, pancreas)	6 (14.3)
	
*Number of metastases*	
1	28 (66.7)
2	6 (14.3)
⩾3	8 (19.1)

**Table 2:** Tumour response (intention-to-treat analysis)

**Response**	**Number (*n*=42, %)^a^**
Confirmed response	19 (45.2)^a^
Complete response	1 (2.3)
Partial response	18 (42.9)
Stable disease	12 (28.6)
Progressive disease	8 (19.1)
Not assessable	3 (7.1)

a 95% Confidence interval=31.7–59.7%.

**Table 3:** Adverse reactions

	**Grade^a^ (% of patients, *n*=42)**		**Grade^a^ (% of cycles, *n*=186)**	
	**3**	**4**	**3**	**4**
*Hematologic*				
Anaemia	4.8			
Leukopenia	7.1	4.8	4.8	1.6
Neutropenia	7.1	19.0	12.9	7.5
Thrombocytopenia	2.4		0.5	
				
*Non-hematologic*				
Nausea	2.4		0.5	
Vomiting	4.8		5.9	
				
*Fatigue*				
Stomatitis	2.4		0.5	
Diarrhoea	4.8		1.1	
				
*Peripheral neuropathy* b				
Febrile neutropenia	9.5		2.2	

a National Cancer Institute Common Toxicity Criteria version 3.0.

b According to an oxaliplatin specific scale (Caussanel *et al*, 1990).
